# Unraveling the impact of a germline heterozygous *POLD1* frameshift variant in serrated polyposis syndrome

**DOI:** 10.3389/fmolb.2023.1119900

**Published:** 2023-01-23

**Authors:** Laia Bonjoch, Yasmin Soares de Lima, Marcos Díaz-Gay, Isabella Dotti, Jenifer Muñoz, Leticia Moreira, Sabela Carballal, Teresa Ocaña, Miriam Cuatrecasas, Oswaldo Ortiz, Antoni Castells, Maria Pellisé, Francesc Balaguer, Azucena Salas, Ludmil B. Alexandrov, Sergi Castellví-Bel

**Affiliations:** ^1^ Gastroenterology Department, Institut d’Investigacions Biomèdiques August Pi i Sunyer (IDIBAPS), Centro de Investigación Biomédica en Red de Enfermedades Hepáticas y Digestivas (CIBERehd), Hospital Clínic, Barcelona, Spain; ^2^ Department of Cellular and Molecular Medicine and Department of Bioengineering and Moores Cancer Center, UC San Diego, La Jolla, CA, United States; ^3^ Inflammatory Bowel Disease Unit, Gastroenterology Department, Institut d'Investigacions Biomèdiques August Pi i Sunyer (IDIBAPS) Centro de Investigación Biomédica en Red de Enfermedades Hepáticas y Digestivas (CIBERehd), Hospital Clínic, Barcelona, Spain; ^4^ Pathology Department, Institut d’Investigacions Biomèdiques August Pi i Sunyer (IDIBAPS), Centro de Investigación Biomédica en Red de Enfermedades Hepáticas y Digestivas (CIBERehd) and Tumor Bank-Biobank, Hospital Clínic, Barcelona, Spain

**Keywords:** serrated polyposis syndrome, POLD1, genetic predisposition to disease, gene editing, functional genomics

## Abstract

Serrated polyposis syndrome (SPS) is one of the most frequent polyposis syndromes characterized by an increased risk for developing colorectal cancer (CRC). Although SPS etiology has been mainly associated with environmental factors, germline predisposition to SPS could also be relevant for cases with familial aggregation or a family history of SPS/CRC. After whole-exome sequencing of 39 SPS patients from 16 families, we identified a heterozygous germline frameshift variant in the *POLD1* gene (c.1941delG, p.(Lys648fs*46)) in a patient with SPS and CRC. Tumor presented an ultra-hypermutated phenotype and microsatellite instability. The *POLD1* germline variant segregated in three additional SPS-affected family members. We attempted to create yeast and cellular models for this variant but were no viable. Alternatively, we generated patient-derived organoids (PDOs) from healthy rectal tissue of the index case, as well as from a control donor. Then, we challenged PDOs with a DNA-damaging agent to induce replication stress. No significant differences were observed in the DNA damage response between control and *POLD1*-Lys648fs PDOs, nor specific mutational signatures were observed. Our results do not support the pathogenicity of the analyzed *POLD1* frameshift variant. One possible explanation is that haplosufficiency of the wild-type allele may be compensating for the absence of expression of the frameshift allele. Overall, future work is required to elucidate if functional consequences could be derived from *POLD1* alterations different from missense variants in their proofreading domain. To our knowledge, our study presents the first organoid model for germline *POLD1* variants and establishes the basis for its use as a model for disease in SPS, CRC and other malignancies.

## 1 Introduction

Serrated polyposis syndrome (SPS) is a clinically defined syndrome characterized by multiple serrated polyps in the colon and rectum as well as an increased risk for developing colorectal cancer (CRC) (Carballal et al., 2022; Muller et al., 2022). Traditionally, serrated polyps were often missed during colonoscopy, and there was a lack of awareness regarding their malignancy potential as precursor lesions of CRC. Currently, SPS is the most common colorectal polyposis syndrome, although its underlying causes are still unclear. SPS has been mainly associated with environmental factors such as smoking and alcohol consumption (Bailie et al., 2017). However, it has also been hypothesized that SPS likely represents a spectrum of disease influenced by genetic and environmental factors (Mankaney et al., 2020). Germline predisposition to SPS could be relevant for those cases with familial aggregation or a family history of either SPS or CRC.

Studies attempting to identify the genetic basis of SPS have provided inconsistent data so far. *RNF43* is the gene with more robust evidence of being involved in SPS predisposition (Gala et al., 2014; Taupin et al., 2015; Yan et al., 2017; Mikaeel et al., 2022; Murphy et al., 2022), although its association is quite controversial and is now believed to be a minor germline cause of SPS (Buchanan et al., 2017; Quintana et al., 2018). Polyposis-related genes are not commonly altered in SPS patients (Clendenning et al., 2013); but some isolated SPS cases harbor putatively pathogenic variants in *MUTYH* (Chow et al., 2006; Boparai et al., 2008; Murphy et al., 2022), *SMAD4, CHEK2* and *POLD1* (Murphy et al., 2022). Our research group has conducted germline sequencing analyses and has proposed new potential high-penetrance genes (Toma et al., 2020; Soares de Lima et al., 2021; 2022) and low-penetrance variants (Arnau-Collell et al., 2020) that could be associated with SPS susceptibility.

The high-fidelity polymerases epsilon (*POLE)* and delta (*POLD1*) have a selective polymerase active site coupled with a proofreading exonuclease domain, essential for an effective DNA repair activity during DNA replication. The alteration of their proofreading activity results in the accumulation of mutations throughout the genome, which leads to a high mutational burden and specific mutational signatures (termed, COSMIC signatures SBS10a-d) that can be identified by whole-exome or whole-genome sequencing (Alexandrov et al., 2020; Robinson et al., 2021). Germline mutations in the proofreading domains of *POLE* and *POLD1* predispose to colorectal adenomas and adenocarcinomas (Palles et al., 2013) but also to extra-intestinal neoplasia such as ovarian, endometrial and brain tumors (Valle et al., 2019; Magrin et al., 2021). *POLE* and *POLD1* can also be somatically mutated in colorectal tumors and, sometimes, mutations in these polymerases can appear in combination with deficiencies in DNA mismatch repair (Haradhvala et al., 2018). Other pathogenic *POLE/POLD1* germline variants, either affecting the catalytic domain or intronic regions, have been associated with growth restriction and multisystem disorders such as IMAGe (intrauterine growth restriction, metaphyseal dysplasia, adrenal hypoplasia congenita and genitourinary abnormalities), FILS (facial dysmorphism, immunodeficiency, livedo and short stature), MDP (mandibular hypoplasia, deafness, progeroid features), or Werner syndrome (Schmit and Bielinsky, 2021).

Organoid technology has revolutionized cancer modeling, and it corresponds to a huge step forward for the study of tumor initiation and cancer progression (Lau et al., 2020). Both patient-derived organoids (PDO), which retain the original mutational background of the patient, or genetically-engineered organoids, hold great promise for hereditary cancers and comprehension of syndromes such as SPS. For instance, pathogenicity for variants in *BRAF* (Fessler et al., 2016) and *RNF43* (Yan et al., 2017; Fang et al., 2022; Yamamoto et al., 2022) has been functionally assessed in these *in vitro* models. Also, the serrated pathway of carcinogenesis has been reproduced in organoids by sequential editing approaches (Lannagan et al., 2018; Kawasaki et al., 2020).

In this study, we have combined whole-exome sequencing with organoid modeling to assess the impact of a germline heterozygous *POLD1* frameshift variant detected in a family with SPS.

## 2 Materials and methods

### 2.1 Patients and clinical samples

The SPS cohort comprised 16 families including 39 patients (≥2 patients per family) diagnosed with SPS and fulfilling the 2010 World Health Organization (WHO) criteria (Snover et al., 2010), as the new WHO guidelines released in 2019 (Rosty et al., 2019) were not available when this study was developed. The complete clinical and somatic characterization of this cohort is available at (Soares de Lima et al., 2021). The presence of germline alterations in *APC*, *MUTYH* and DNA mismatch repair (MMR) genes was discarded for all probands.

**FIGURE 1:**
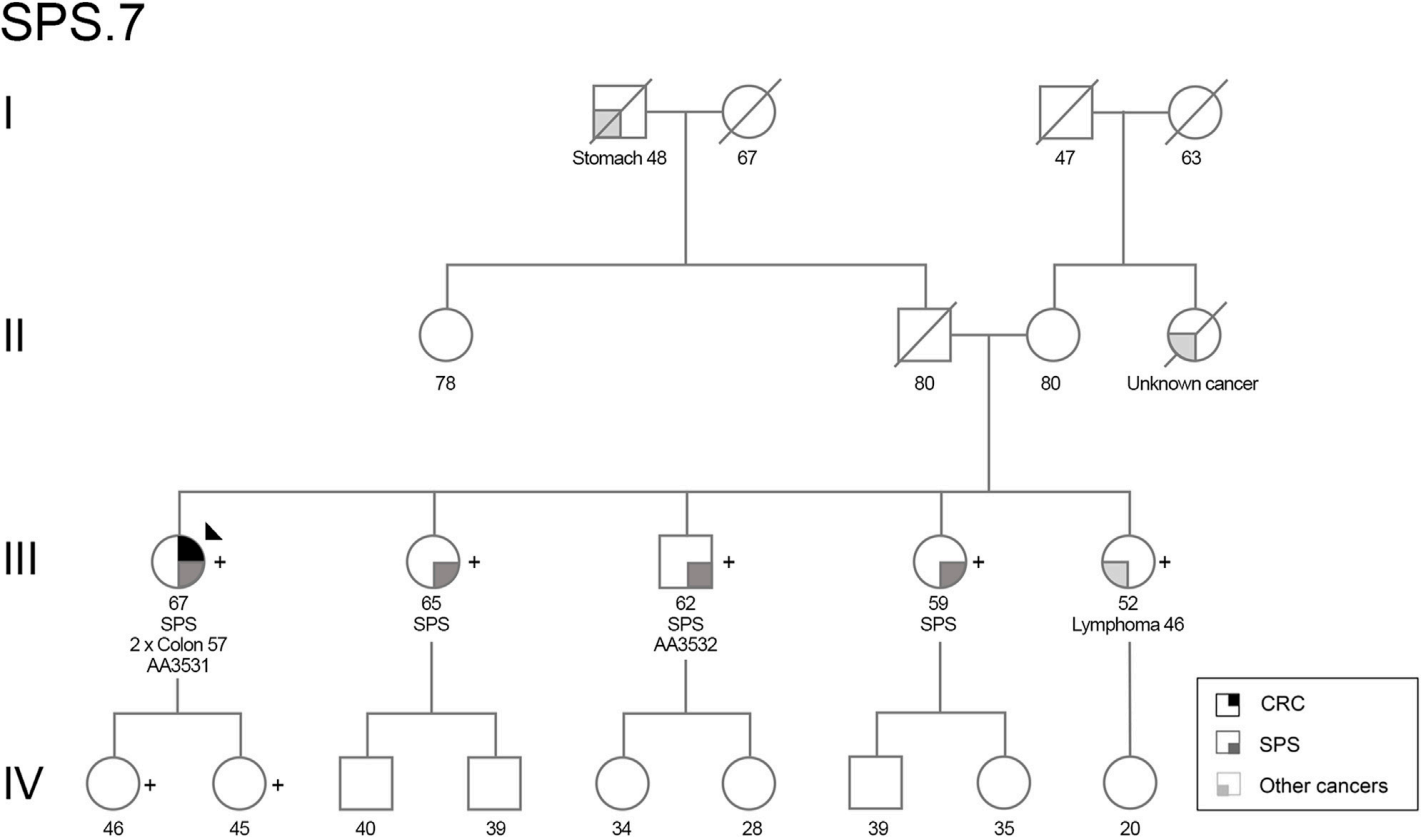
Pedigree of family SPS.7. Filled symbol indicates affected for CRC (upper right quarter), SPS (lower right quarter) or other types of cancer (lower left quarter). Stomach, colon and lymphoma refer to the type of cancer. The proband is indicated by an arrow, and *POLD1* p.Lys648fs*46 variant carriers are indicated by (+). Ages at diagnosis are depicted. CRC, colorectal cancer; SPS, serrated polyposis syndrome.

One patient (AA3531, family SPS.7, Figure 1) presented a loss-of-function variant in the *POLD1* gene (c.1941delG; p.Lys648fs*46). The variant co-segregated in other six family members, three of them affected with SPS (Figure 1; Supplementary Figure S1). A summary of clinical characteristics of family SPS.7 is shown in Table 1.

**TABLE 1:** Clinical characteristics of family SPS.7.

Patient	Gender	SPS	SPS 2010 criteria	Polyps (N)	CRC
AA3531 (III.1)	F	Y	1 + 3	>100 serrated polyps	Y
III.2	F	Y	2	5 T/LGD adenomas; 6 serrated polyps	N
AA3532 (III.3)	M	Y	2	15 T/LGD adenomas; 5 serrated polyps	N
III.4	F	Y	2	7 T/LGD adenomas; 4 serrated polyps	N
III.5	F	N		0	N*
IV.1	F	N		Unknown	N
IV.2	F	N		Unknown	N

SPS, serrated polyposis syndrome; N, number; CRC, colorectal cancer; T, tubular; LGD, low-grade dysplasia. *diagnosed with MALT, lymphoma.

The index case (III.1) was affected with 2 synchronous CRC at age 57 as well as more than 100 serrated polyps, and important proportion of them having a large size (>20 mm). Individual III.5 was diagnosed at 46 y. o. with MALT lymphoma (affecting gastrointestinal tract, breast and lung). A familial history of cancer was present with a paternal grandfather affected with stomach cancer at 48 y. o. and a maternal aunt diagnosed with cancer of unknown origin at 78 y. o.

Regarding the phenotype in the affected siblings, III.2 presented 11 polyps at 56–65 y. o., corresponding to one hyperplastic polyp in the rectum (3 mm), five sessile serrated lesions proximal to the rectum (5–8 mm) and 5 T/LGD (tubular, low-grade dysplasia) adenomas (4–8 mm) distributed all over her colon. III.3 presented 20 polyps <1 cm at 53–60 y. o., including 5 serrated polyps proximal to the rectum (5–6 mm), being four hyperplastic polyps and one sessile serrated lesion without dysplasia, and 15 T/LGD adenomas (<1 cm). III.4 presented 11 polyps at 49–57 y. o., comprising four serrated polyps being two sessile serrated lesions proximal to the rectum (>5 mm, <1 cm), and 7 T/LGD adenomas (one 1 cm at rectum, the rest <1 cm).

The study received the approval of Hospital Clínic de Barcelona Clinical Research Ethics committee (registration number 2013/8286). Written informed consent was obtained in all cases.

### 2.2 Whole-exome sequencing, variant identification and prioritization

Details on germline and tumoral whole-exome sequencing, quality control and alignment, variant calling and variant annotation have been already described in (Soares de Lima et al., 2021).

### 2.3 DNA extraction and amplification

Germline DNA and cell lines’ DNA was extracted using the QIAamp DNA Blood kit (Qiagen, Redwood City, CA, United States). Somatic DNA was obtained from formalin-fixed paraffin-embedded tissue using the QIAamp Tissue kit (Qiagen, Redwood City, CA, United States). *POLD1* variant validation was performed by PCR amplification using the GC-Rich PCR System (Roche, Basel, Switzerland) followed by Sanger sequencing (Eurofins Genomics).

### 2.4 Somatic characterization

#### 2.4.1 Microsatellite instability

Tumor MMR deficiency (MMRd) was evaluated by immunohistochemical staining of the four mismatch repair proteins (MLH1, MSH2, MSH6 and PMS2). MSI (microsatellite instability) status was assessed by analyzing five nearly monomorphic mononucleotide microsatellite loci (BAT-25, BAT-26, NR-21, NR-24, and MONO-27; Promega, Madison, WI).

DNA methylation status of the *MLH1* promoter region was established by bisulfite genomic sequencing, as previously described (Moreira et al., 2015).

#### 2.4.2 POLD1 immunohistochemistry

Immunostainings were performed on histological 2-μm sections. After deparaffination, antigen retrieval was performed with citrate buffer 10 mM, and tissue was permeabilized with 1% Triton X-100. Peroxidase activity was blocked with 3% hydrogen peroxide. Sections were treated for 1 h with Dako serum-free protein blocker (Agilent, Santa Clara, CA), incubated for 16 h with anti-POLD1 antibody (EPR15118, #ab186407, Abcam) diluted 1:500, and for 1 h with goat anti-rabbit secondary antibody at 37°C (Dako REAL EnVision HRP Rabbit; Agilent). Sections were revealed with diaminobenzidine for 10 s (Agilent), counterstained with hematoxylin and mounted. An Olympus BX41 microscope (Olympus, Tokyo, Japan) was used to visualize the immunostainings.

#### 2.4.3 Loss of heterozygosity

Loss of heterozygosity (LOH) was tested by comparing germline-tumoral Sanger sequencing results of the same individual. Additionally, microsatellites mapping close to *POLD1* (D19S866, D19S904, D19S246, D19S907) were assessed by PCR. Primer details are listed in Supplementary Table S1.

#### 2.4.4 Mutational signatures

Assignment of reference mutational signatures was performed using our bioinformatics tool SigProfilerAssignment v0.0.14 (https://github.com/AlexandrovLab/SigProfilerAssignment/) (Islam et al., 2022) based on COSMIC mutational signatures v3.3 (GRCh37 genome build) (Tate et al., 2019).

### 2.5 RNA extraction from blood

Whole blood was collected into PAXgene Blood RNA tubes (PreAnalytiX, Hombrechtikon, Switzerland), and automated purification of total RNA was performed using the QIAcube and the PAXgene Blood RNA Kit (Qiagen), according to the manufacturer’s protocol.

Total RNA from cultured cells was isolated with the RNeasy Mini Kit, according to the manufacturer’s instructions (Qiagen, Hilden, Germany).

### 2.6 PBMCs isolation

Peripheral Blood Mononuclear Cells (PBMCs) were isolated from whole blood by density gradient centrifugation (Ficoll^®^ Paque, GE Healthcare Life Sciences). Ten ml of whole blood were mixed 1:1 with PBS, layered on top of 3 ml of density gradient media, and separated by centrifugation at 1800 rpm for 25 min at room temperature with the brake turned off. After recovering the buffy coat, PBMCs were washed three times with cold PBS. Pelleted PBMCs were finally cryopreserved for subsequent protein expression testing.

### 2.7 Quantitative real time-PCR

RNA reverse transcription was performed with the High-Capacity cDNA reverse Transcription kit (Applied Biosystems). Quantitative PCR was run on a QuantStudio1 System (Applied Biosystems) by using Taqman^®^ Gene Expression probes against POLD1-FAM (Hs01100821_m1) and GAPDH-VIC (4326317E), the latter for normalization purposes. Relative quantification was performed with the –∆∆Ct method.

### 2.8 Protein extraction and immunoblotting

Pelleted PBMCs or cultured cells were lysed with RIPA buffer solution (Sigma-Aldrich, MA, United States) supplemented with cOmplete Protease Inhibitor Cocktail and PhosSTOP (Roche, Basel, Switzerland).

Protein extracts were run in NuPAGE™ gels according to the manufacturer’s protocol (ThermoFisher, Waltham, MA) and transferred into PVDF membranes (Millipore, Bedford, MA). Blots were probed with anti-POLD1 (EPR15118, #ab186407, Abcam) or anti-GAPDH (clone 14C10, #2118, Cell Signaling) primary antibodies diluted 1:5000, followed by the incubation with the fluorescent Dylight 800 anti-rabbit secondary antibody (SA5-10036). Protein detection was carried out using Odyssey Imaging System (LI-COR, Lincoln, NE).

### 2.9 Cell lines

Human colorectal SW837 cells (diploid and MMR-proficient, Cat No. 91031104, ECACC, Sigma Aldrich) were cultured in RPMI-1640 medium (Gibco, Waltham, MA) supplemented with 10% fetal bovine serum. Human HEK293T cells (Cat No. CRL-3216, ATCC) were cultured in DMEM medium (Gibco, Waltham, MA) supplemented with 10% fetal bovine serum. Both cell lines were maintained under standard growth conditions (37°C, 5% CO_2_) and routinely tested for *mycoplasma* contamination using the *Mycoplasma* Gel Detection kit from Biotools (Madrid, Spain).

### 2.10 Development of a *POLD1* ^+/−^ cellular model by CRISPR/Cas9 gene editing

The Benchling (http://benchling.com) CRISPR tool was used to design suitable single guide RNAs (sgRNA) and homology-directed repair (HDR) templates flanking the *POLD1* p.Lys648 region. Additionally, as a positive control, we designed a sgRNA and HDR template to model the *POLD1* p.Leu474Pro mutation, a pathogenic founder mutation present in Spanish population (Accession ClinVar: VCV000144003.5) (Valle et al., 2014; Ferrer-Avargues et al., 2017).

sgRNA top and bottom strands were purchased from IDT (Coralville, IA) and cloned into the Esp3I site of the lentiCRISPRv2-Puro plasmid (#98290, Addgene), which also packages the Cas9 coding sequence. Each lentiCRISPRv2-*POLD1* encoding vector was packaged into lentivirus by using the host cell line HEK293T and the CalPhos Mammalian Transfection kit (TakaraBio, Kusatsu, Japan). HEK293T cells were plated and co-transfected with at a 3:2:1 lentiCRISPRv2:psPAX2:pVSVG2 DNA ratio. Supernatants were harvested at two different time points (24 and 48 h after transfection), pooled, concentrated by centrifugation (15,000xg, 3 h, 4°C) and used for cell transduction in the presence of 8 μg/ml of polybrene. Infected cells were enriched by puromycin selection (1 μg/ml).

Cells with a stable expression of the sgRNA and the Cas9 protein were plated at a ratio of 400,000 cells per well in 12-well plates. Cells were transiently transfected in two consecutive rounds with a mixture of 10 pmol (500 ng) of the HDR template and 125 ng of an episomal vector for p53DD (#25989, Addgene) by using Lipofectamine 3000 reagent (ThermoFisher, Waltham, MA). The dominant negative p53DD inhibits the P53 double-strand break (DSB) response and was used to boost the DNA engineering process (Ihry et al., 2018). Cells were seeded into 96-well plates at a density of 1 cell per well and after 3 weeks, the obtained clones were screened for *POLD1* gene editing by Sanger sequencing (Eurofins Genomics).

### 2.11 Cell viability assessment

Cell viability was determined using the colorimetric CellTiter 96^®^ AQueous One Solution Cell Proliferation kit (Promega, Madison, WI). Either SW837 or SW837-*POLD1* ^+/−^ cells were seeded in 96-well plates at a density of 2500 cells per well, in triplicate. After 4 days, 20 μl of CellTiter reagent was added to each well. Plates were incubated at 37°C for 3 h and absorbance was read at 490 nm wavelength using an Epoch Microplate Spectrophotometer (BioTek, Winooski, VT).

### 2.12 Clonogenic assay

Single-cell suspensions were seeded at low density (400 cells per well into 12-well plates). After 16 days, colonies were fixed in cold methanol for 10 min and stained with a 0.5% crystal violet solution (Sigma Aldrich). After drying, plates were imaged on an EliSpot Reader System (AID GmbH, Strassberg, Germany) and analyzed with ImageJ (National Institutes of Health, Bethesda, MD).

### 2.13 Organoid culture establishment

The organoid cultures used in this study were established from normal rectum endoscopic biopsies derived from patient AA3531:III-1 (Figure 1) and a healthy donor, following already published procedures (Dotti et al., 2022).

### 2.14 γH2AX immunofluorescence assessment

Organoids were expanded in µ-Slide 8-well ibiTreat chambers (Ibidi, Fitchburg, WI) and treated with 200 nM camptothecin (CPT) for 24 h. As a control, some wells were left untreated. Phosphorylation of the Ser-139 residue of the histone variant H2AX (γH2AX) was assessed by immunofluorescence staining following already published protocols (Mayorgas et al., 2021), with some modifications. After the fixation step, organoids were stored in PBS overnight at 4°C, and the next day the permeabilization and blocking steps were performed as indicated. Samples were then incubated with anti-γH2AX (#ab81299, 1:750, Abcam) and EpCAM (#M0804, 1:150, Dako) primary antibodies overnight at 4°C. The next day, samples were incubated with the secondary antibodies anti-rabbit Alexa Fluor^®^ 594 and anti-mouse Alexa Fluor^®^ 488 (ThermoFisher, Waltham, MA), both diluted 1:500. After DAPI nuclear staining, samples were overlaid with Ibidi Mounting medium (#50001, Ibidi, Fitchburg, WI) and stored at 4°C for subsequent fluorescent microscope observation in a Zeiss LSM 880 confocal laser scanning microscope (CCiTUB optical microscopy facility, Universitat de Barcelona, Spain). Positive nuclei (γH2AX–DAPI colocalization) were counted by using the CellCounter plug-in in ImageJ software. The ratio of γH2AX-positive nuclei *versus* the total number of nuclei per organoid was calculated.

### 2.15 Organoid somatic mutational profile

Organoids were expanded in 48-well plates and treated with 200 nM CPT for 24 h. After the genotoxic challenge, organoids were cultured during a 5-day resting period to allow cells to accumulate mutations. Organoids were recovered, and DNA extraction was performed in order to assess changes in their mutational profile, as already mentioned in section 2.4.4. Somatic variant calling was performed using MuTect2 (McKenna et al., 2010) and Strelka2 (Kim et al., 2018), by only considering those variants shared by both computational tools and showing an allelic frequency above 0.20.

## 3 Results

### 3.1 Characterization of somatic mutations

**FIGURE 2:**
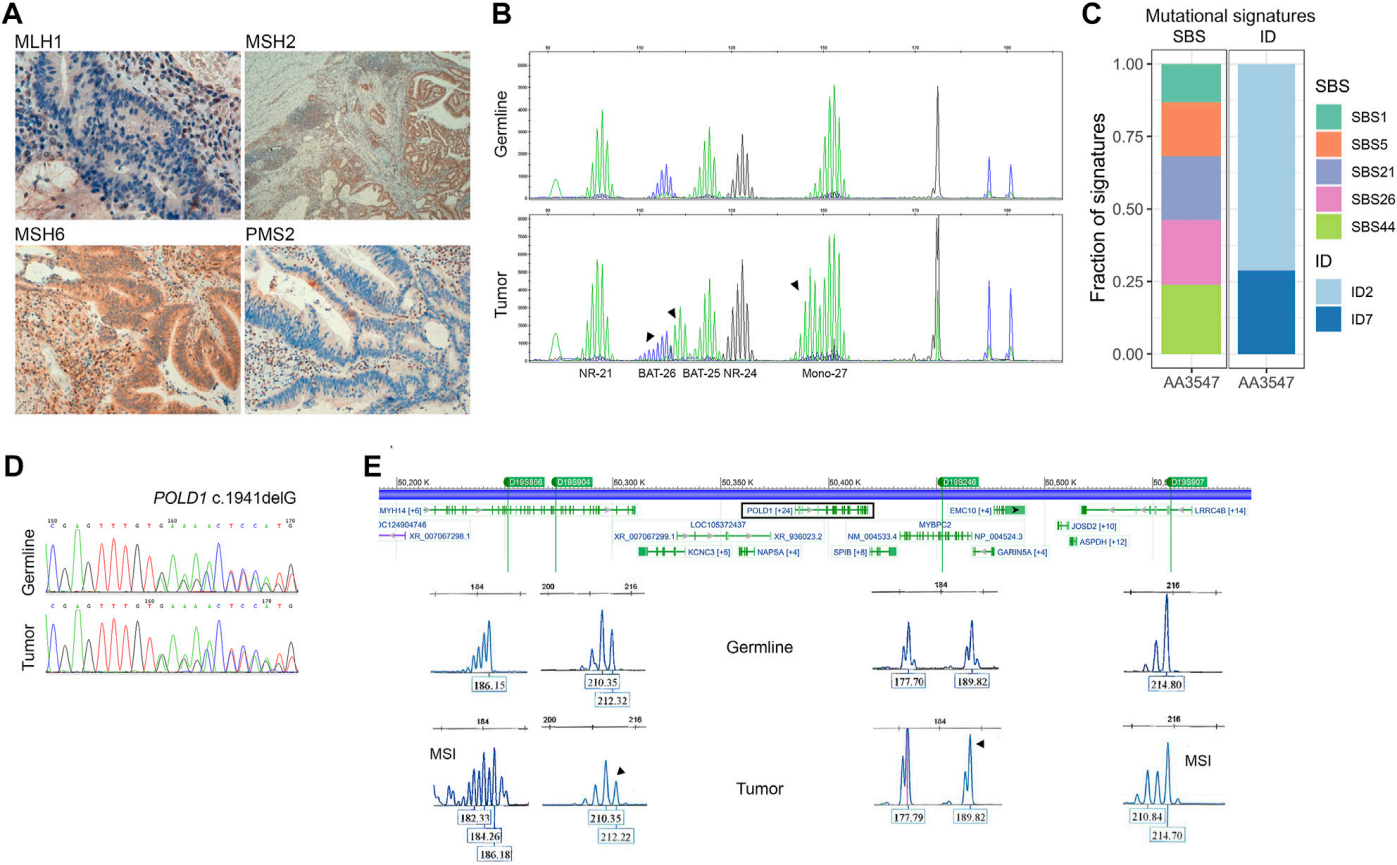
Somatic characterization of the index case. **(A)** Immunohistochemistry for MMR proteins showing loss of MLH1 and PMS2 expression. **(B)** Microsatellite instability analysis in germline DNA (upper panel) and tumor DNA (lower panel). All markers showing instability are marked with an arrow. **(C)** Contribution of single base substitution (SBS) and small insertions and deletions (ID) mutational signatures on a tumoral DNA sample from the proband (AA3547). **(D)** Loss-of-heterozygosity assessment by Sanger sequencing and by **(E)** PCR-amplification of four microsatellite markers close to *POLD1* (rectangle). The four loci D19S866, D19S904, D19S246 and D19S907 are depicted in green. LOH is indicated by an arrow. MSI, microsatellite instability.

Tumor MMRd testing was performed in both tumor samples from the proband. Immunostaining for the four MMR proteins confirmed loss of MLH1 and PMS2 (Figure 2A), and MSI molecular testing revealed that the tumor was indeed MSI-H, due to the alteration of three mononucleotide markers (BAT25, BAT26, Mono-27) (Figure 2B). One tumor also presented *MLH1* promoter hypermethylation (25%), a likely cause for the observed MMR deficiency.

Germline DNA (AA3531) and somatic DNA (AA3547) from one MMRd tumor of the proband underwent whole-exome sequencing for the assessment of mutational signatures, tumor substitution mutational burden (TMB), and tumor indel mutational burden (IDB). The sample displayed the COSMIC clock-like signatures SBS1 and SBS5, and a high contribution of the already described MMRd-associated signatures SBS21, SBS26, SBS44, ID2, and ID7 (Figure 2C), in concordance with the molecular MMRd characterization of the tumor. Neither the mutational signatures associated with a defective *POLD1* proofreading (SBS10c, SBS10d) nor that associated with concurrent *POLD1* mutations and defective DNA mismatch repair (SBS20) were detected. However, the sample appeared to be ultra-hypermutated (TMB = 117.46 mut/Mb, IDB = 100.6 mut/Mb), which is characteristic from combined mismatch-repair deficiency and polymerase alterations.

Additionally, LOH analysis was performed by Sanger sequencing and the detection of microsatellite markers flanking POLD1. The analysis revealed a LOH of the wild-type allele (Figures 2D,E).

### 3.2 *POLD1* variant segregation and expression analysis in the SPS.7 family

**FIGURE 3:**
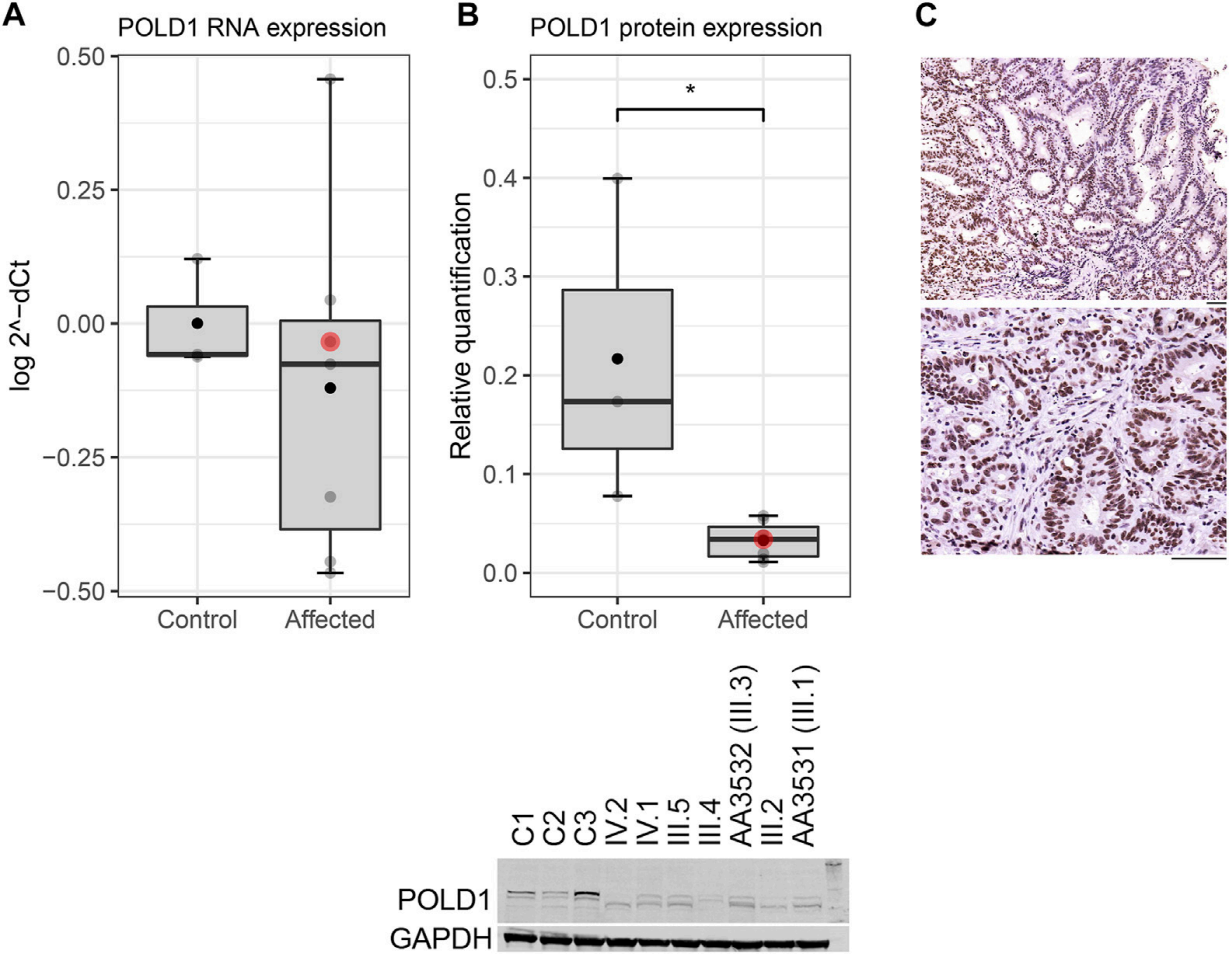
POLD1 expression in *POLD1* p.Lys648fs*46 variant carriers at both **(A)** RNA and **(B)** protein levels. A significant difference of POLD1 protein expression was detected in *POLD1* p.Lys648fs*46 carriers (affected) *versus* healthy controls (Wilcoxon test, *p* < 0.05). The horizontal line marks the median value, and the black dot indicates the mean value. The proband is indicated by a red dot. **(C)** Nuclear detection of POLD1 in two different adenocarcinoma sections from a proband’s tumor sample. Scale bars, 50 µm.

The segregation analysis revealed that the *POLD1* variant c.1941delG, p.(Lys648fs*46) segregated in six additional members of the family beside the index case: four siblings (three fulfilling SPS 2010’s criteria and one diagnosed with lymphoma at age of 46) and her two healthy daughters (Figure 1; Supplementary Figure S1). The variant is located in the polymerase domain of *POLD1* and it causes a frameshift, which changes a Lysine to an Arginine at codon 648, and a premature stop codon is predicted at position 46 of the new reading frame (Supplementary Figure S2A). We evaluated the germline expression of this gene at both RNA and protein levels in the identified variant carriers. We observed a decrease on POLD1 levels (Figures 3A,B), suggesting that the altered mRNA was being degraded by the non-sense mediated decay pathway rather than producing a truncated protein. Nevertheless, although the germline protein expression pattern was markedly reduced in the Western Blot, the nuclear detection of POLD1 in the proband’s tumor revealed that POLD1 expression from the wild-type allele was still evident (Figure 3C).

### 3.3 CRISPR/Cas9 generation of a *POLD1* ^+/−^ model

We planned to establish a *POLD1* p.(Lys648fs*46) model in SW837 cells using CRISPR/Cas9 *via* homologous recombination. At the same time, we also intended to introduce the founder pathogenic mutation *POLD1* p.Leu474Pro as a positive control. We tested three different CRISPR strategies: the transient transfection of the sgRNA and Cas9 cloned into a plasmid together with the HDR template; the transient nucleofection of the sgRNA and Cas9 as a ribonucleoprotein complex together with the HDR template; and the generation of a cellular model constitutively expressing the sgRNA and Cas9, in which the HDR template was transiently transfected. The high on-target efficiency of Cas9 and the high mortality observed in all the attempts confirmed *POLD1* as an essential gene. After several targeting rounds, we failed to generate a heterozygous model encoding the *POLD1* p.(Lys648fs*46) variant. However, one of the attempts with the last strategy randomly produced a clone with a different frameshift mutation and a premature stop codon (Supplementary Figure S2A). Although far from ideal, we pursued to functionally characterize this clone, termed hereafter *POLD1* ^+/−^ for convenience.

*POLD1* ^+/−^ cells showed POLD1 downregulation at both RNA and protein levels, similar to what was observed in *POLD1* p.(Lys648fs*46) carriers (Supplementary Figures S2B, 2C). We next assessed whether a reduced amount of wild-type POLD1 could be important for cell viability. Cell proliferation and cell survival were not affected in *POLD1* ^+/−^ cells, indicating that reduced POLD1 levels did not affect cellular growth (Supplementary Figures S2D, 2E). We also challenged *POLD1* ^+/−^ cells with different concentrations of the DNA-damaging agent CPT, but no significant differences were observed between wild-type SW837 cells and *POLD1* ^+/−^ cells, suggesting that a single *POLD1* functional copy allele could maintain normal function (Supplementary Figure S2F).

### 3.4 Patient-derived organoids

**FIGURE 4:**
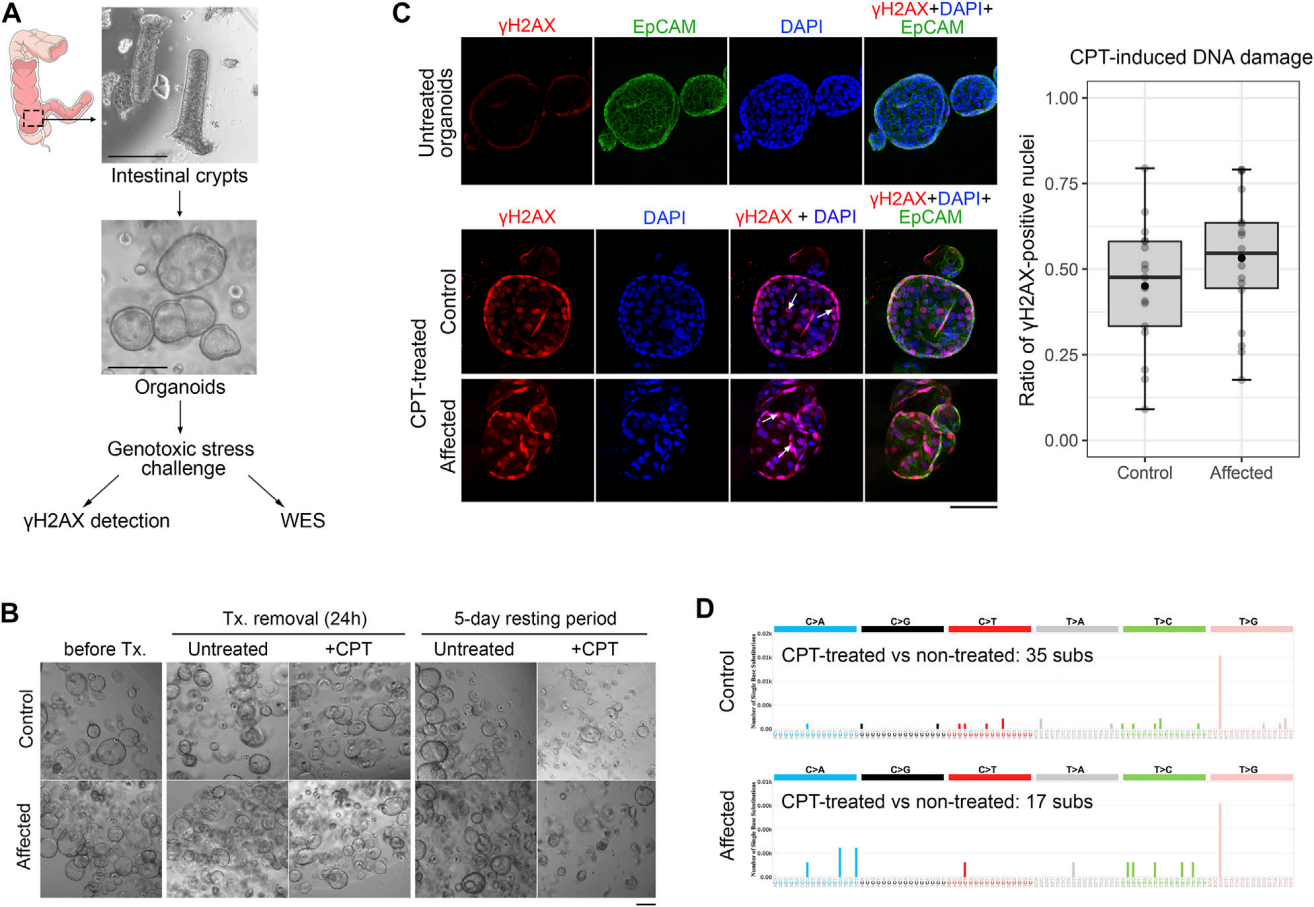
Generation of PDO and DNA damage and repair functional assessment in a *POLD1* p.Lys648fs*46 carrier. **(A)** Overview of the PDO generation and functional characterization workflow. Endoscopic samples from the index case (*POLD1* p.Lys648fs*46 carrier, Affected) and a healthy control were used to generate PDO. Organoids were subsequently exposed to CPT in order to induce genotoxic stress and evaluated for DNA damage (γH2AX) and DNA repair (WES) after a 5-day resting period. Scale bars, 200 µm. **(B)** Representative images of PDO from control and *POLD1* p.Lys648fs*46 carrier (Affected) at different stages of the experiment. Panel scale bar, 200 µm. **(C)** DNA damage assessment after CPT treatment by γH2AX immunofluorescence staining. γH2AX-DAPI colocalization (white arrows) was assessed and γH2AX-positive nuclei were quantified. EpCAM counterstaining was also performed. Untreated organoids showed negligible γH2AX signal (representative image of control organoids is displayed). Panel scale bar, 100 µm. **(D)** DNA repair assessment by WES after CPT-treatment and a 5-day resting period. The profiles of the mutational signatures are depicted. CPT, camptothecin; Tx, treatment; WES, whole-exome sequencing.

To study the specific functional consequences of *POLD1* p.(Lys648fs*46) variant, we generated patient-derived organoids (PDOs) from normal rectum biopsies from the proband, which maintained the genetic background of the original tissue and, therefore, already had the loss-of-function *POLD1* variant in their genome (*POLD1* ^K648fs^). At the same time, PDOs from a control individual were also generated. PDOs were stimulated with CPT to evaluate the effect of *POLD1* haplosufficiency in both DNA replication and DNA damage repair (Figure 4A). After the genotoxic stress challenge, we aimed to assess the amount of DNA damage by γH2AX immunofluorescence staining, as it has emerged as a highly specific and sensitive molecular marker for monitoring DNA damage initiation and resolution (Mah et al., 2010). After a 24-h treatment, organoid growth and shape were unaffected (Figure 4B). The increase in γH2AX phosphorylation upon CPT treatment was evident in both control and *POLD1* ^K648fs^ organoids, but no differences in the amount of γH2AX positive nuclei per organoid were observed between them (Figure 4C), indicating that reduced POLD1 expression did not alter the organoid sensitivity to CPT. We next assessed whether the CPT-induced DNA damage repair could be impaired in organoids lacking a functional copy of POLD1. After a 5-day resting period, the organoid growth arrest was evident in both control and *POLD1* ^K648fs^ organoids (Figure 4B). The DNA from CPT-treated organoids and their untreated counterparts was collected, and whole-exome sequencing was performed. The number of substitutions detected in the samples was very low, and the mutational profiles did not indicate any accumulation of drug-induced mutations (Figure 4D).

## 4 Discussion

Currently, it is still controversial whether SPS’s origin could have an underlying genetic predisposition. Although smoking and other environmental exposures have been associated with SPS development, germline predisposition factors could still be relevant, especially in cases with familial aggregation.

In this study, we assessed the impact of the *POLD1* p.(Lys648fs*46) variant detected by whole-exome sequencing in a proband with familiar history of SPS and cancer. Although one of the tumors from the proband displayed a MMRd profile, it also showed LOH and ultra-hypermutation, which is usually correlated with *POLE/POLD1* alterations. At the same time, the detected *POLD1* frameshift variant segregated in three additional family members with serrated polyposis. Therefore, the role of *POLD1* p.(Lys648fs*46) alteration in the germline predisposition to SPS in this family remained to be assessed.

Several studies have focused on the effect of *POLE/POLD1* germline variants located in the exonuclease domain (Elsayed et al., 2015; Bellido et al., 2016; Buchanan et al., 2018; Mur et al., 2020a; Siraj et al., 2020), but limited efforts have been made to determine the functional impact of those variants identified in the polymerase domain. Their potential pathogenicity is usually based on rarity of these variants in the general population and loss-of-function intolerance scores. Nevertheless, functionally relevant mutations could also occur outside the *POLD1* exonuclease domain, leading to defects in nucleotide selectivity and error-prone polymerase activity (Daee et al., 2010; Mertza et al., 2015; Barbari and Shcherbakova, 2017). Also, a decrease in POLD1 protein levels seems to reduce the efficiency of both replicative DNA synthesis and DNA synthesis associated with DNA repair, probably due to an increased frequency of DNA polymerase slippage (Kokoska et al., 2000; Lemoine et al., 2008; Tumini et al., 2016). Since both POLD1 quantity and quality seem important for genome stability, frameshift/non-sense heterozygous variants’ functional implications could also be hypothesized.

In order to determine the functional consequences of the *POLD1* p.(Lys648fs*46) variant, we proceeded to generate a suitable cellular model. Traditionally, yeast-based models have been used to assess the pathogenicity of *POLD1* variants due to the high homology between the human and yeast polymerase delta catalytic subunit. We first intended to model *POLD1* p.(Lys648fs*46) in *Schizosaccharomyces pombe* using ade6-485 reversion, a strategy we and others have previously used to validate missense variants of *POLE* and *POLD1* (Palles et al., 2013; Esteban-Jurado et al., 2017; Mur et al., 2020a). However, *POLD1* is an essential gene, and *S. pombe* strains are haploid, which hampers the modeling of non-sense/frameshift variants in this model. Therefore, we pursued editing *S. pombe* in its diploid state. After several attempts and high mortality rates, the model could not be established, as diploid strains are unstable and difficult to propagate in the laboratory.

The CRISPR/Cas9 technique offers a new approach to potentially edit any desired genome region. Nevertheless, the engineering of *POLE* and *POLD1* in mammalian cells has been scarce and mainly performed in MMRd cell lines (Hodel et al., 2020; Job et al., 2020). In our case, the CRISPR-driven *POLD1* editing was a major challenge in the MMR-proficient SW837 cell line and could not be accomplished for *POLD1* p.(Lys648fs*46). The randomly obtained *POLD1* ^+/−^ model allowed us to corroborate that cells expressing a single *POLD1* copy had reduced expression levels of POLD1 at both RNA and protein levels, similar to what was observed in *POLD1* p.(Lys648fs*46) carriers. Although POLD1 reduction or depletion had been previously linked to cell cycle arrest and increased sensitivity to the DNA damaging agents CPT, hydroxyurea (Tumini et al., 2016) and methyl methane sulfonate (Kokoska et al., 2000), cellular growth and sensitivity to CPT were not altered in our cellular model.

The generation of PDOs from normal tissue of the proband allowed us to characterize the effect of the *POLD1* frameshift variant on DNA damage and repair responses. *POLD1* ^K648fs^ and control organoids showed an equivalent DNA damage sensitivity to CPT treatment, in line with what was observed in the *POLD1* ^+/−^ cellular model. Also, we did not detect specific mutational signatures associated with DNA repair malfunction after a 5-day resting period. However, it should be considered that we did not perform clonal organoid culturing and expansion for a long time period, which could influence these results. Organoids have proven to be a suitable model in which mutational signatures can be associated with genetic defects. Using CRISPR-edited colon organoids, it was possible to confirm the mutational signatures associated with *NTHL1* and *MLH1* loss-of-function (Drost et al., 2017). Also, the mutational signatures associated with *POLE*/*POLD1* exonuclease domain defects have been reproduced *in vitro* in tumor PDOs with a *POLE* hotspot mutation (Yan et al., 2020), *POLE* CRISPR-edited cells (Hodel et al., 2020) and *POLD1* patient-derived fibroblasts (Andrianova et al., 2022). To our knowledge, this is the first time that *POLD1* variants are functionally assessed by organoid modeling.

Altogether, our results do not support the pathogenicity of *POLD1* heterozygous non-synonymous/frameshift variants. It could be hypothesized that haplosufficiency of the *POLD1* wild-type allele compensates for the altered allele, a mechanism already reported in heterozygous *pol3*-exo^−^ yeast mutants (Zhou et al., 2021) and heterozygous germline *POLD1* p.Leu474Pro carriers (Andrianova et al., 2022). However, the origin of the ultra-hypermutated TMB detected in the proband remains elusive since only a hypermutated profile will be expected from MMRd. Neither the tumoral sample nor CPT-challenged *POLD1* ^K648fs^ organoids displayed signatures SBS10c-d or SBS20, linked to *POLD1* exonuclease malfunction alone or in conjunction with MMRd. In a similar reported case, in which a patient with a *POLE* frameshift germline variant presented mainly somatic MMRd-associated signatures, it could not be concluded whether the identified variant increased colorectal cancer predisposition (Yamaguchi et al., 2019; Mur et al., 2020b; Yamaguchi and Furukawa, 2020). It has also been speculated that *POLD1* pathogenic variants cause hypermutation only with concurrent MMRd, with the latter as an early event (Schamschula et al., 2022). However, it should also be considered whether mutational signatures associated with *POLD1* exonuclease domain malfunction could differ from those arising from loss-of-function alterations.

In summary, our results do not support the pathogenicity of *POLD1* frameshift variants, and we hypothesize that *POLD1* could be an essential gene that exhibits haplosufficiency. To our knowledge, our study presents the first organoid model for germline *POLD1* variants. Overall, it is still unclear if functional consequences could be derived from *POLD1* alterations different from missense variants in their proofreading domain.

## Data Availability

The original contributions presented in the study are included in the article/Supplementary Material, further inquiries can be directed to the corresponding authors.
