# Supplementary material for: Unraveling the impact of a germline heterozygous POLD1 frameshift variant in serrated polyposis syndrome
Source: Front Mol Biosci. 2023 Jan 23;10:1119900. doi: 10.3389/fmolb.2023.1119900 (PMC9900627; doi:10.3389/fmolb.2023.1119900)
Supplement: Supplementary file 1 [file DataSheet1.pdf]

Supplementary Material

Unraveling the impact of a germline heterozygous *POLD1* frameshift variant in serrated polyposis syndrome

Laia Bonjoch*, Yasmin Soares de Lima, Marcos Díaz-Gay, Isabella Dotti, Jenifer Muñoz, Leticia Moreira, Sabela Carballal, Teresa Ocaña, Miriam Cuatrecasas, Oswaldo Ortiz, Antoni Castells, Maria Pellisé, Francesc Balaguer, Azucena Salas, Ludmil B. Alexandrov, Sergi Castellví-Bel*

*** Correspondence:** Dr. Laia Bonjoch and Dr. Sergi Castellví-Bel: bonjoch@recerca.clinic.cat; sbel@recerca.clinic.cat

Supplementary Table 1. List of primers used in this study.

	**Forward (5'-3')**	**Reverse (5'-3')**
***POLD1* variant validation**		
POLD1 p.Lys648fs*46	GCACAGGCCCAGAGATAGT A	TGAGCAGGTTCTCCAGGAT
***POLD1* LOH**		
D19S866	CATGAGTTTGACTATGAAGA CG	CACTCCAGCCTGGGTAA
D19S904	ACAAGAATTGCTTGAACCTG G	GCTCCATTTCGGAGATGTTA
D19S246	AGAGTGAGATTCCACCTTTC	GAAACACATCATTTACCCAC
D19S907	GTGTCCAATCAACAGACCA	CTGCACTCCAGCAGAAAT

Supplementary Table 1 (cont.)

**CRISPR guides and homology recombination templates**		
POLD1 p.Lys648fs*46 sgRNA	GGTCTTCACAAACTCGTCCC	
POLD1 p.Leu474Pro sgRNA	GCTCACGGCATTGAGCGTGT	
POLD1 p.Lys648fs*46 ssODN	GTAGGGCAGAGGTGGGCTGGAGCAGGAGGGTGGCCGGCAGTCACCCCAACATCTTCCAACCCAGCCTGACTGAGGATCAGTTCATCAGGACCCCCAC**a**GGGGACGA**a**TT**c**GTAAGACCTCAGTGCGGAAGGGGCTGCTGCCCCAGATCCTGGAGAACCTGCT	
POLD1 p.Leu474Pro ssODN	GGGGTGTGTCCCTGTCCTTGGAAGGCCACTGCCCAGGCCCGCAGCCCACCAGCCCACCCACCCACCTAGGTGCTGCTGCGGGAGTACAAGCTCCG**g**TC**t**TACACGC**c**CAATGCCGTGAGCTTCCACTTCCTGGGCGAGCAGAAGGAGGACGTGCAGC	
**Gene editing validation**		
POLD1 p.Lys648fs*46	TGTTACACCACGCTCCTTCGGC	ACACGGGGAAAGCACACTCAGG
POLD1 p.Leu474Pro	TCCTCAGGTACAAACATTCCC	CCTTGTCTCTGCTTTCCTGG

LOH, loss-of-heterozygosis; ssODN, single-stranded oligodeoxynucleotides

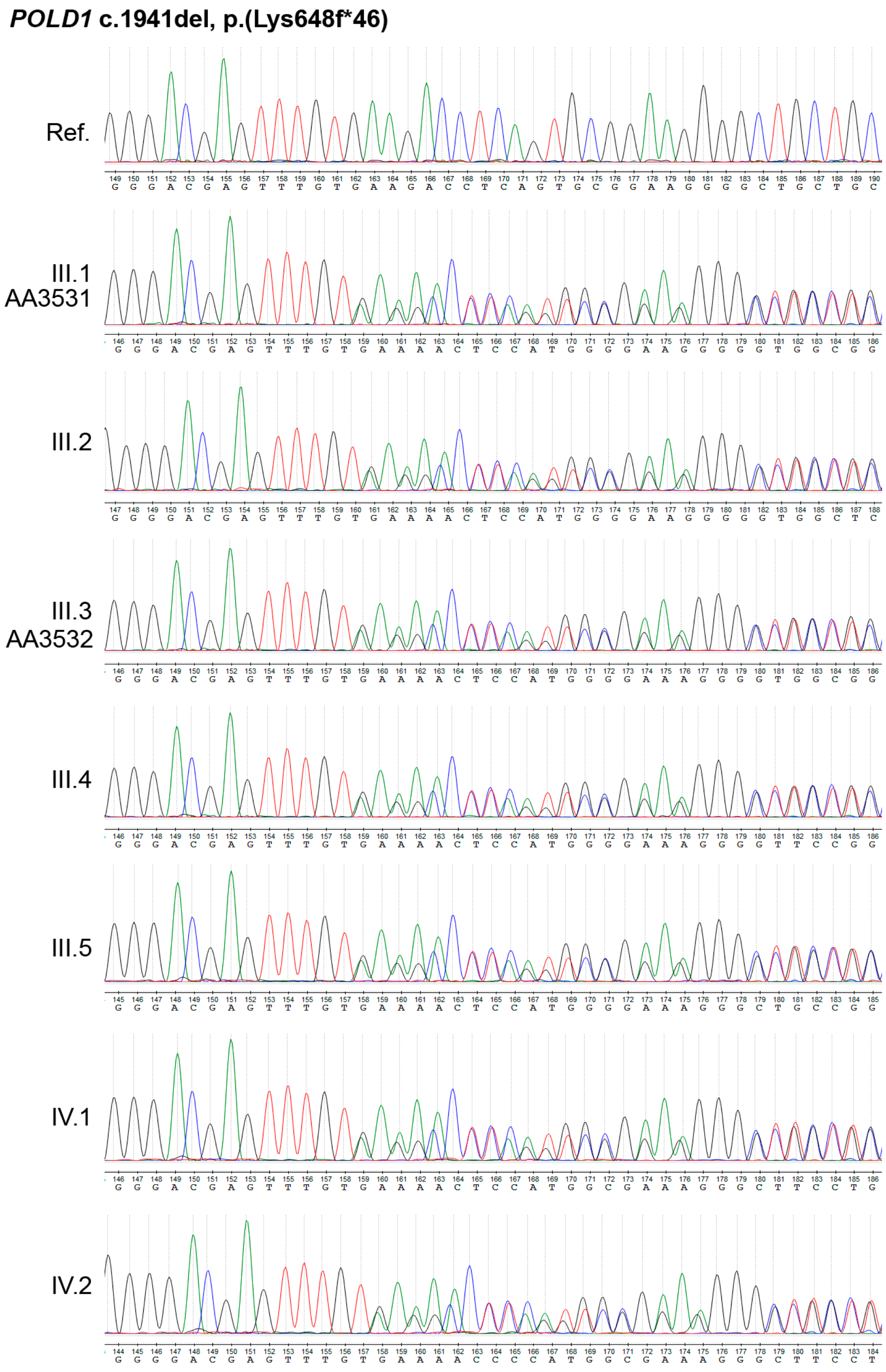

Supplementary Figure 1. *POLD1* p.Lys648fs*46 variant validation and segregation analysis. A reference sequence from a noncarrier is also presented.

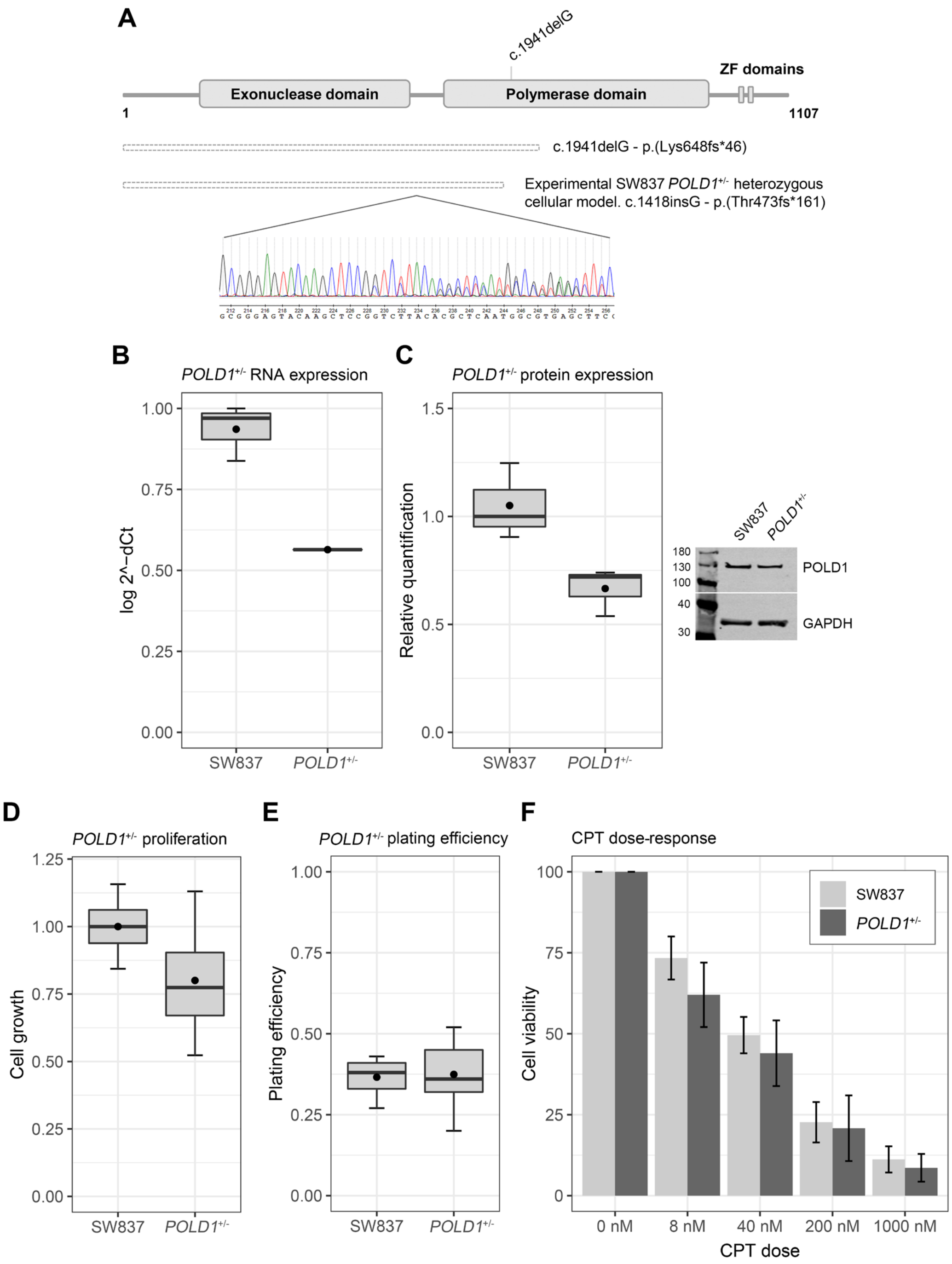

Supplementary Figure 2. *POLD1*$^{+/-}$ cellular model generated by CRISPR/Cas9 gene editing in SW837 cells. **(A)** Mapping of the *POLD1* p.Lys648fs*46 variant within the functional domains of the protein. The expected protein product is depicted and compared with the randomly generated *POLD1*$^{+/-}$ heterozygous model. **(B)** POLD1 RNA and **(C)** protein expression in *POLD1*$^{+/-}$ cells. **(D)** MTS cell proliferation assay. **(E)** Plating efficiency (colonies originated from single cells) in the colony formation assay. **(F)** Dose-response curve and cell survival assessment after a 4-day CPT treatment. In all box plots, the horizontal line marks the median value, and the black dot indicates the mean value. Samples were assayed in triplicate and the experiments were repeated three times (n = 3). CPT, camptothecin; ZF, zinc fingers.
